# Restless Legs Syndrome across the Lifespan: Symptoms, Pathophysiology, Management and Daily Life Impact of the Different Patterns of Disease Presentation

**DOI:** 10.3390/ijerph17103658

**Published:** 2020-05-22

**Authors:** Giuseppe Didato, Roberta Di Giacomo, Giuseppa Jolanda Rosa, Ambra Dominese, Marco de Curtis, Paola Lanteri

**Affiliations:** 1 Clinical and Experimental Epileptology and Sleep Disorders Unit, Foundation IRCCS Carlo Besta Neurological Institute, Via Celoria 11, 20133 Milan, Italy; roberta.digiacomo@istituto-besta.it (R.D.G.); rsogpp@unife.it (G.J.R.); ambra.dominese@istituto-besta.it (A.D.); marco.decurtis@istituto-besta.it (M.d.C.); 2 Neurology Unit, University Hospital of Ferrara, 44100 Ferrara, Italy; 3 Neurophysiopathology Unit, Foundation IRCCS Carlo Besta Neurological Institute, 20133 Milan, Italy; paola.lanteri@istituto-besta.it

**Keywords:** restless legs syndrome, sleep, lifespan, age, quality of life, workplace, gender, genetic, environment

## Abstract

Restless legs syndrome is a common but still underdiagnosed neurologic disorder, characterized by peculiar symptoms typically occurring in the evening and at night, and resulting in sleep disruption and daily functioning impairment. This disease can affect subjects of all age ranges and of both sexes, manifesting itself with a broad spectrum of severity and deserving special attention in certain patient categories, in order to achieve a correct diagnosis and an effective treatment. The diagnosis of restless legs syndrome can be challenging in some patients, especially children and elderly people, and an effective treatment might be far from being easy to achieve after some years of drug therapy, notably when dopaminergic agents are used. Moreover, the pathophysiology of this disorder offers an interesting example of interaction between genetics and the environment, considering strong iron metabolism involvement and its interaction with recognized individual genetic factors. Therefore, this syndrome allows clinicians to verify how lifespan and time can modify diagnosis and treatment of a neurological disorder.

## 1. Introduction

Restless legs syndrome (RLS), also known as Willis–Ekbom disease, is a frequent neurological disorder whose recognition among neurologists is still low, despite the typical symptoms reported by patients [1]. This condition was first described in 1685 by Sir Thomas Willis, a British anatomist and physician, but it was in 1944 that Karl Axel Ekbom, a Swedish physician, reported all the clinical features and coined the term RLS [2]. Patients typically present with sensory symptoms and discomfort in their legs, an urge to move at rest above all in the evening, and consequently, sleep disturbances, but they are often undiagnosed and untreated for years [3,4]. Disease manifestation is determined by complex environmental and genetic interactions, rendering RLS an interesting model to study how genes interact with the environment to express themselves [5]. RLS can occur as an isolated disease, mostly at a young age, or can be associated with comorbidities such as cardiovascular disease, diabetes, renal failure, arterial hypertension and peripheral neuropathy [6]. The role of peripheral nerve damage in the causality and severity progression for RLS patients remains unclear [7]. However, the subdivision of RLS in two entities, primary or secondary to another disease, has recently become a matter of debate, after the discovery of genetic data and studies of hypoxic pathway activation and iron deficiency, providing further insights into the pathophysiology of the disease [8]. The better knowledge of RLS pathophysiology gives outstanding contribution to the treatment of the disease. Even though dopaminergic treatment can be an effective first-line therapy, long-term dopaminergic therapy, especially levodopa, which has a short half-life, is related to the occurrence of the so-called augmentation. Although patients are recommended to receive the lowest effective dose for the shortest period of time, some patients are in fact under a stable condition for years with a lower dose of long acting dopamine agonists. Different treatment options must be frequently used during the evolution of the disease, such as pregabalin, gabapentin, oxycodone–naloxone, and iron preparations, but most patients report inadequate long-term management of symptoms [9].

RLS is a disease uniquely showing how lifetime of the patients generally or of a single patient as well can be interested in different ways. RLS can affect people all across their lifespan, existing early-onset forms of the disease and late-onset forms as well. Different periods of a lifetime can be differently affected, depending on how the symptoms manifest themselves at various ages (e.g., children versus adults or elderly people) and in distinct life conditions (e.g., pregnancy or other physiological or pathological events interacting with RLS pathophysiology) or at different ages of a single patient [10]. Comorbidities appearing overtime can complicate or modify the way the disorder presents itself, as well as can represent the main cause of the symptoms [6]. The pathophysiology of the disease, although consisting in some main mechanisms, can be modified or enriched by some cofactors, peculiar for different age ranges or for specific conditions (e.g., pregnancy), thus, contributing to symptomatology [11]. Quality of life of the patients is differently affected, according to the subject’s age and social and work commitments [12]. Moreover, the management of the disease, in the same patient, shows different problems developing overtime, since disease progression, tolerance or augmentation usually appear during the course of this disorder, especially when it is treated with dopaminergic agonists. Therefore, drug therapy of RLS changes across the lifespan of a patient, requiring specific adjustments in specific conditions [13].

## 2. Epidemiology

RLS prevalence has been estimated between 5% and 10% in Caucasian populations, above all European and North American people, while in Asian countries, prevalence is lower. However, if only clinically significant RLS is taken into account (i.e., 1–2 times per week, moderate to severe distress), a 2% to 3% prevalence is reported. Noteworthy, prevalence in women is twice as high as in men, and elderly people are more affected than children and adults: prevalence increases up to 60–70 years of age [14,15].

Children are less affected, but pediatric RLS does exist and prevalence is estimated around 2% in the pediatric population. In younger children, the description of symptoms is difficult, and thus, RLS can be underreported. Therefore, RLS prevails among school-aged children and adolescents (2%–4%), who are more prone to show clinically significant RLS [15,16].

According to age at first symptoms, early-onset (younger than 45 years) and late-onset (older than 45 years) RLS can be distinguished. Early-onset RLS is often familial and has a slow progression of symptoms, while late-onset RLS may show a rapid evolution and is more frequently associated with comorbidities, above all iron deficiency [17].

## 3. Clinical Features and Diagnosis of RLS

RLS is a sensorimotor disorder characterized by typical symptoms with a peculiar circadian way of presentation. The patients complain of a strong, irresistible urge to move the legs, often accompanied by uncomfortable sensations deeply in the legs. The urge typically begins or worsens during periods of inactivity (e.g., lying down or sitting) and are relieved by movement, such as walking or stretching. Symptoms occur in the evening or at night and can progressively worsen across the night, but tend to resolve spontaneously by early hours of the morning [15,17,18].

Patients may have difficulty describing their symptoms, often using various terms such as “restless”, “twitchy”, “need to stretch”, “urge to move”, “tension”, “itching”, “burning” and “legs want to move on their own”. The RLS sensations are often described as painful, but isolated pain with an urge to move is uncommon and should raise the suspicion that the patient may not have RLS [15,17].

RLS symptoms usually are located distal to the knee in the calf region, but the symptoms can also affect other body parts, such as the thigh and the arms. Most patients describe that they perceive the discomfort deep inside the leg and less commonly superficially. The symptoms generally involve the limbs bilaterally, but they can also alternate on the two sides or more rarely be strictly unilateral [18].

The patient’s complaints may be present daily or occasionally, and a day-to-day variability occurs, so that a distinction between intermittent RLS (symptoms less than twice weekly for the past year) and chronic persistent RLS (at least twice weekly for the past year) can be done [17,18].

Despite the peculiarity of the clinical presentation of the disease, a great number of patients is still unrecognized or, worse, misdiagnosed by general practitioners and by neurologists [1]. To address the problem of the underdiagnosis of RLS, new diagnostic criteria have been established by a consensus of RLS experts from the International RLS Study Group (IRLSSG) in 2014 [18]. Moreover, the American Academy of Sleep Medicine published a slightly modified version in their International Classification of Sleep Disorders (ICSD-3) [15].

The IRLSSG criteria point above all on the presence of five clinical features (Table 1). Therefore, the diagnosis is based on the subjective description of symptoms by the patient and no instrumental examination (e.g., polysomnogram) is strictly required to confirm the diagnosis [18].

However, an overnight polysomnogram can be useful to quantify the periodic leg movements of sleep (PLMS), which are stereotyped leg movements, occurring in about 90% of RLS patients. They are characterized by extension of the big toe with partial flexion of the ankle, knee and sometimes the hip, usually bilaterally but not always synchronously [19]. Upper limbs can occasionally be involved [20]. These movements can also occur while awake, and they are called periodic limb moments during wakefulness (PLMW) [21].

PLMS can be used as an accessory diagnostic criterion, along with response to dopaminergic treatment, especially in difficult to diagnose RLS patients. They may give information on RLS severity and on the amount of sleep instability and fragmentation, because PLMS are typically associated with an increase in heart rate and blood pressure, and often also to an arousal [18]. This could explain the mechanism by which RLS can increase the risk of developing cardiovascular disease, even though there is no clear-cut evidence of an association between RLS and cardiovascular risk [22].

Differential diagnosis with other disorders affecting the legs must be taken into consideration when asking the patient, such as arthritis, leg cramps, peripheral neuropathy, venous stasis, myalgia or leg edema (Table 2) [17,18]. The patient’s age, sex, professional risks, and comorbidities can represent factors focusing the differential diagnosis, but confounding conditions may exist if the same patient suffers from multiple pathologies [6]. Akathisia can be observed in patients taking neuroleptics, but it must be noted that these drugs can induce RLS symptoms as well. As opposed to RLS, akathisia is characterized by absence of a circadian pattern and by restlessness, which is not associated with unpleasant sensations or sensory discomfort localized in the legs. In order to relieve their restlessness, the patients usually show whole-body involvements, sometimes body-rocking movements or marching in place, but these movements often are not effective in reducing the urge to move [23,24].

Primary or idiopathic RLS occurs when the patient does not suffer from any other disorder that can be considered a potential cause of RLS symptoms. Patients with medical or neurological diseases could also have RLS, but the term secondary RLS, sometimes used in these instances, should be limited to the only few diseases with a proven association with RLS, such as iron deficiency anemia and uremia. However, by looking into the phenomenology of these cases of secondary restless legs syndrome, it is apparent that there is no difference in the clinical symptomatology or therapeutic response compared with primary forms of the disorder [1,6].

With regard to clinical significance, the main consequence of RLS is sleep disruption. Hence, the more the sleep is disturbed, the more the disorder will have a clinical impact, in terms of cognitive functions and quality of life [18]. Sleep latency and arousal index are typically increased in RLS patients who do not nevertheless report significant excessive daytime sleepiness, but rather daily fatigue [18].

Moreover, considering that sleep is an essential component of human health, suboptimal sleep duration has been associated with greater burden of disease, increased cardiovascular and metabolic morbidity, immune and hormone dysfunction and mortality [25,26].

Among secondary RLS, an important and not uncommon form is iatrogenic RLS. Some drugs can provoke RLS or aggravate it if already present. Therefore, a detailed recollection of the patient’s drug history is mandatory, including over-the-counter medications. The main implicated drugs are: antihistamines, above all sedating antihistamines, often used as hypnotics, such as diphenhydramine; neuroleptics/dopamine-receptor blockers (e.g., olanzapine, risperidone, quetiapine); serotonergic and noradrenergic antidepressants (e.g., amitriptyline, fluoxetine, sertraline, venlafaxine, mirtazapine), which can worsen both PLMS and RLS symptoms; anti-emetics (e.g., metoclopramide). Antidepressants, such as trazodone or bupropion, which have dopaminergic activity, should be preferred because they do not provoke or exacerbate RLS symptoms [17,27,28].

## 4. Pathophysiology and Genetics

RLS is characterized by a strong familial clustering, with up to 63% of patients having at least one first-degree relative suffering from the same disorder, especially for the early-onset form. This suggests the presence of genetic factors determining the pathology, and an autosomal dominant pattern of inheritance is usually observed in familial studies [17].

Iron deficiency and dopamine metabolism are the two main pathophysiological mechanisms underlying RLS symptoms, but a link to genetics does exist.

Some genome-wide association studies (GWAS) have identified RLS risk alleles on five specific genomic regions (MEIS1, BTBD9, PTPRD, MAP2k/SKOR1, and TOX3/BC034767) and on an intergenic region on chromosome 2 (rs6747972). Most of these variants show an association to the PLMS and one of them, a risk allele on BTBD9, is also strongly associated with decreased serum ferritin, i.e., decreased peripheral iron stores. MEIS1, a gene implicated in limb development, shows the strongest association to RLS, raising the possibility that RLS may also be caused by a developmental disorder. MEIS1 is one of the most promising genes and seems to be important for the regulation of brain iron homeostasis. However, there are probable additional genetic contributors yet to be identified [17,29,30,31,32].

Iron pathophysiology has been extensively studied. It is based on the observation that decreased peripheral iron and iron-deficient anemia are strongly associated with RLS. Moreover, conditions that alter iron status, such as pregnancy and renal failure, are risk factors for RLS, whose symptoms are relieved by treatment of iron deficiency [5,8].

However, normal serum ferritin is found in most RLS patients, suggesting the presence of possible different pathophysiological mechanisms involving iron metabolism. Studies conducted in patients with normal peripheral iron stores have led to the hypothesis that central nervous system iron status could be more important in RLS. Indeed, reduced cerebrospinal fluid (CSF) ferritin and decreased brain iron have been reported in RLS patients with normal serum ferritin. Brain iron deficiency has been shown in neuropathological specimens and on magnetic resonance imaging (MRI) involving, above all, the substantia nigra and, to a lesser extent, the putamen, the caudate and the thalamus [8,33,34].

This regional brain iron deficiency is linked to a decreased activity of the *iron regulatory protein-1* on the microvessels, a protein involved in the transport and storage of iron into the brain, specifically into the cells of the substantia nigra. Therefore, also in the presence of a normal peripheral iron status, a decreased iron intake and storage in specific brain regions can occur with a reduction in the transferrin receptor on the endothelial cells of the blood–brain barrier [5,8,35,36].

The main consequences of brain iron deficiency are hypoxia and myelin loss. Iron regulates oxygen transport at the level of microvessels towards the neural cells, and decreased iron should potentially activate a hypoxic pathway. The latter may have a role in the induction of an altered dopaminergic activity, as it will be explained below [5,8].

Regional iron deficiency affects also myelin synthesis, which depends on iron. Therefore, the brain iron deficiency of RLS can produce a mild myelin deficit in the corpus callosum, anterior cingulum, and precentral gyrus, confirmed by brain imaging and postmortem analyses. This myelin deficit could contribute to RLS symptoms by altering the sensorimotor integration [37].

The dopamine pathophysiology of RLS finds its fundamentals in the discovery of the dramatic response to dopaminergic agents (first levodopa, in the early 1980s, then dopamine agonists), implying a likely dopaminergic brain deficiency [5,17]. However, some studies have shed light on a more complex and partly surprising series of pathophysiological mechanisms involving dopamine metabolism in the brain. The level of the dopamine metabolite homovanillic acid (HVA) in the CSF has been found to be increased in RLS patients, suggesting an increased dopamine production, that is, counterintuitively, a hyperdopaminergic state [38].

Brain imaging studies produced results that, although partly contradictory, indicate increased striatal dopamine, instead of the expected decrease. The nuclear medicine studies, positron emission tomography (PET) and single-photon emission computerized tomography (SPECT), found a reduction in striatal D2 receptors, which suggests a response to increased synaptic dopamine [39,40]. The fluoro-L-dopa (fDOPA) studies have shown decreased striatal fDOPA uptake which, in the absence of cell loss in RLS, supports a fast turnover of dopamine consistent with increased dopamine production [41]. As for dopamine transporter (DAT) imaging, SPECT studies found normal total DAT, but decreased membrane-bound DAT, as in increased striatal dopamine [42].

There seems to be, thereby, an apparent contradiction because of RLS symptom response to the administration of dopamine agonists in the presence of an excess of brain dopamine. This can be explained taking into account that increased dopamine induces a postsynaptic downregulation of striatal D2 receptors and of intracellular function [42,43]. However, dopamine has a physiological circadian activity, with a peak in the morning and a minimum in the evening and at night. The morning increase of dopamine activity can be sufficient to compensate the postsynaptic downregulation, but later, during the daytime a dopaminergic activity deficit may arise, and this can trigger RLS symptoms [5]. The hyper-alertness typically observed in the morning in RLS patients can be related to the same aforementioned mechanism, preventing sleepiness even after a short and disrupted sleep.

A small dose of dopamine in the evening and night can correct the relative evening decrease in dopamine, but this leads to increased downregulation, thus, worsening RLS symptoms and causing the so-called augmentation. This initially will require higher dopamine agonist doses to be effective and can resemble tolerance to the drug, but it finally turns out to be a worsening of the underlying disease pathophysiology [5,13].

How the dopamine metabolism instability translates into the typical RLS symptoms still remains a matter of debate. Some hypotheses are: (1) the alteration of a descending hypothalamic dopaminergic pathway (the A11 region cell group in the dorsoposterior hypothalamus), projecting to the spinal cord and acting as inhibitory system on the spinal roots [44,45,46]; (2) imbalance of the intraspinal dopaminergic receptors, D3 in the dorsal horn (sensory symptoms) and D1 in the anterior horn (motor symptoms), also related to altered descending dopaminergic pathways [1,47]; (3) increased sensorimotor cortex excitability, shown by transcranial magnetic stimulation (TMS) studies of the motor cortex [48,49], as a consequence of altered striatal and non-striatal dopaminergic transmission. Impaired somatosensory integration could therefore be present. The last hypothesis may also be related to iron deficiency, because of myelin and white matter alterations, revealed by MRI [37], after brain iron deficits.

Moreover, iron and dopamine pathophysiological mechanisms seem to be related one to another, since the hypoxic pathway, activated by brain iron imbalance, interferes with dopamine metabolism, activating the enzymatic cascade producing an increased dopamine synthesis [8]. It follows that distinguishing primary from secondary RLS on the basis of iron deficiency could be debatable, since iron is involved in the pathophysiology even when iron deficiency is not evident. On the other hand, some authors include iron deficiency in the definition of primary RLS because of its essential role in the disease genesis [8,50].

Regarding hypoxia, the probable relationship with RLS is based also on the practical observation of high prevalence of RLS symptoms in subjects exposed to decreased blood oxygen saturation (people residing at high altitudes or patients with chronic pulmonary disease). In addition, decreased oxygen concentrations and microvascular endothelial changes in the legs of patients with RLS have been described [1]. Indeed, hypoxia and hypoxic pathway activation have been found also in the leg muscles of RLS patients, as a consequence of a likely generally altered iron metabolism. An increased difference between morning and evening blood flow and hypoxia in leg muscles of RLS patients has been suggested [51]. Hypoxia activates intracellular mechanisms, possibly involving nitric oxide and adenosine, which in turn, induce transcriptional processes, leading to an increase in dopamine synthesis, among other alterations.

Moreover, it has been shown that, in addition to dopamine, adenosine as well as other neurotransmitters such as glutamate are involved in RLS pathophysiology and this would lead to new treatments acting on these systems [52]. For example, perampanel (a selective α-amino-3-hydroxy-5-methyl-4-isoxazolepropionic acid (AMPA) glutamate receptors antagonist) has shown efficacy in some RLS patient series [53].

## 5. RLS in Women and Pregnancy

As previously mentioned, RLS is more prevalent in women than in men, especially in multiparous women as compared to nulliparous women [14,15,54]. Nulliparous women up to 64 years show the same RLS prevalence described in men of the same age. Therefore, pregnancy seems to be the main factor determining the female predominance of RLS, in addition to the contribution of menses to latent iron deficiency [54].

RLS symptoms typically show a clear peak during the third trimester, frequently recovering after the delivery. Some women who report RLS symptoms during pregnancy have already experienced them before pregnancy, but in most of them, the symptoms appear for the first time during pregnancy [55,56].

There are different hypothetical mechanisms underlying RLS symptoms during pregnancy, the main being the hormonal and metabolic mechanism [11].

The hormonal hypothesis is based on the observation that women using estrogen-based therapies are more prone to develop RLS outside pregnancy than non-estrogen users [57]. Moreover, women developing RLS during pregnancy show increased estradiol levels compared with non-RLS pregnant women [58]. Lastly, the high incidence of RLS symptoms during the third gestational trimester might be related to estrogens, which reach the highest plasmatic levels in late pregnancy.

The metabolic hypothesis depends on the increased iron and folate requirements during pregnancy. Their serum levels decrease across pregnancy, because of fetal needs or hemodilution [11]. It has been shown that iron and folate levels, even though within the normal range, are lower in pregnant women presenting RLS symptoms as compared with pregnant women without RLS complaints [59]. However, these data are controversial, since iron, ferritin and folate serum levels are often normal, and cerebrospinal fluid values of iron and folate, more consistently reflecting the CNS metabolic condition, have never been measured during pregnancy [11].

Other possible mechanisms, in addition to the ones previously mentioned, have also been proposed: (1) Genetic predisposition to RLS (positive family history of RLS in these women), while pregnancy would act as a facilitating condition [56]; (2) Sleep apnea, which is frequent at the end of pregnancy, might trigger RLS; (3) Fetal growth and compression of the nerve roots is considered a further potential contributing factor for RLS during pregnancy; (4) Water retention in the legs can exacerbate RLS symptoms [11,56].

Regarding the consequences of gestational RLS, pregnant patients with RLS show a higher risk of postpartum depression, even though pre-pregnancy depression has been linked to RLS development during pregnancy. Chronic sleep loss, caused by RLS during pregnancy, is the main factor determining the consequences observed in this population: perinatal depression, gestational diabetes, hypertension and preeclampsia, and preterm birth. Moreover, women who suffered from RLS during pregnancy have a 3 to 4-fold increase of the risk of developing RLS later in life, compared with women without gestational RLS [11].

## 6. Children and Elderly People

Considering that no objective tests or clinical biomarkers are needed for the diagnosis of RLS, according to the RLS diagnostic criteria [18], it is apparent that the identification of the disease can be far more difficult in particular age groups, such as children and elderly people.

In pediatric patients, the importance of symptom description in the child’s own words is crucial and, when decision for treatment has to be taken, the clinical course criteria (intermittent vs. chronic RLS) cannot be applied. Children frequently describe their urge to move the legs by means of expressions such as “want to move”, “need to move” or “got to kick”. Therefore, the concept of urge needs an interpretation correlated to the patient’s ability to express him/herself [16,18,60].

The presence of PLMS (with an index of more than 5 per hour) and family history of RLS and periodic leg movement disorder can be supportive criteria, although an overnight polysomnogram is not always available for pediatric patients. It must be noted, however, that in some children PLMS may appear before subjective symptoms and they are found in 63%–74% of children with RLS [60].

As in adults, sleep disruption provoked by RLS affects mood, cognition and function, but in children behavioral problems as well as educational impairment are more prominent as compared to the problems commonly described in adult patients [16,60].

Moreover, for differential diagnosis in pediatric patients, it is necessary to take into account specific conditions, more typical of young patients. Common mimics of RLS in children are positional discomfort, sore leg muscles, growing pains, and tendon strain: if the patient’s complaints are not adequately investigated, an underdiagnosis or a misdiagnosis of RLS in children is possible (Table 2) [23,60].

Regarding growing pains, their co-occurrence with RLS in children in not uncommon, although an overlap between the two conditions has been observed. The diagnostic criteria of growing pains share similar features of RLS, apart from the urge to move and relief of symptoms after movement, more typical of RLS. Moreover, familial clustering of the two disorders and history of growing pains in adults with RLS have been described, and growing pains may benefit from similar drug therapies for RLS (e.g., calcium channel α2δ ligands). Therefore, when leg pain is the main complaint of a child, the two disorders should be considered, not only as a differential diagnosis [61].

Treatment of RLS in children requires special attention, because most drugs used for adults are not approved for children or the experience is limited.

In elderly patients with RLS two main problems can be present: (1) cognitive impairment, causing a difficult identification of the symptoms; (2) comorbidities, far more common in this patient population, thus complicating diagnosis and treatment as well [6,26].

In the case of cognitive decline, the impaired description of symptoms by the patient him/herself often requires the need to rely on objective data, such as the patient behavior or the polysomnographic data, when available. However, the presence of increased PLMS and/or PLMW in senior patients, unless combined with iron deficiency, is not always a good indicator of RLS, because the PLM index, both in wakefulness and in sleep, tends to increase physiologically with age [18].

## 7. Comorbidities and Quality of Life

The clinical impact of RLS is not only related to the fatigue induced by sleep disruption and subjectively perceived by the patient, but it is possibly also linked to a more extended systemic disruption. It has been shown that RLS is associated with different pathologies, not only neurological diseases (migraine, treated Parkinson’s disease) and other sleep disorders, but also cardiovascular disease, hypertension and diabetes [6,12].

The Sleep Heart Health Study [62] showed that the odds ratio for cardiovascular disease was increased in the RLS population to 2.07. An association has been shown between RLS and hypertension, and a mediator might be PLMS, which usually induce sleep instability and an increase in heart rate and blood pressure, especially when PLMS are associated with electroencephalogram arousals. The increase in blood pressure and heart rate leads to a higher sympathetic drive and this consequently, as observed in non-dipping patients (i.e., who do not show a blood pressure decrease at night), raises the risk of developing cardiovascular disease [12,63].

In addition, inflammation is considered another possible mechanism leading to a higher risk for hypertension and cardiovascular disease, because increased C-reactive protein levels have been shown in RLS patients with PLMS. A correlation between C-reactive protein levels and PLMS frequency has been found [64].

Regarding sleep disorders, sleep disruption is reported by the majority of RLS patients, who complain of sleep initiation and sleep maintenance difficulties, decreased total sleep time, and daytime fatigue. Polysomnogram can document prolonged sleep onset latency, shorter total sleep time, higher sleep fragmentation, and reduction in both non-rapid eye movement (NREM) and rapid eye movement (REM) sleep [18,65].

Moreover, up to 31% of RLS patients report episodes of night eating, with a compulsion or “urge” to eat similar to the urge to move the legs typical of RLS. A sleep-related eating disorder (SRED) can thereby be associated with RLS, causing weight gain and an increase of body mass index of these patients. The night eating and SRED may be increased by treatment with sedative hypnotics but not with dopaminergic therapy [66,67].

Quality of life has been shown to be strongly affected by moderate to severe RLS, especially in underdiagnosed patients [12,68]. Multiple studies have documented a reduction in quality of life parameters to a similar degree of patients with chronic medical conditions such as type 2 diabetes mellitus, obesity, depression, and osteoarthritis. Sleep disruption is likely one of the main contributors to the impaired quality of life of RLS patients, compromising physical functioning and cognitive functions during the day as well as mood status, leading to possible depressive symptoms. The latter, together with sleep disturbances associated with RLS, turn to be a mediator of the daily and social disfunction [68].

The social impact of the disease, especially when it is underdiagnosed, has been related to productivity loss at work and to the economic consequences of the possible costs associated with the comorbidities or with RLS itself [68].

The detailed assessment of RLS symptom severity, in parallel with sleep quality, daytime sleepiness, daytime function and mood, and the correct diagnosis and treatment of the disease lead to a clear-cut improvement of quality of life indicators [12].

## 8. Non-Pharmacological and Pharmacological Treatment

RLS pharmacologic treatment is currently based only on symptomatic agents, since a disease-modifying therapy does not exist yet. Non-pharmacologic treatments are also available, although less effective [50].

Nonpharmacologic treatment options have also been suggested, alone for mild cases or associated with pharmacologic therapy. Pneumatic compression of the legs, near-infrared light spectroscopy (NIRS), lifestyle changes (sleep hygiene improvement, change in caffeine or alcohol intake or smoking), yoga, massage, hot baths, aerobics/lower body training, and cognitive behavior therapy have been considered. However, there is insufficient evidence for the majority of these treatments, and they can, in most cases, support the pharmacologic treatments [50,69].

Considering the pathophysiology of the disease, systemic iron deficiency always has to be sought for because iron supplementation alone may relieve RLS symptoms. This is valid also in the case of worsening symptoms in patients on stable therapy, who can show low peripheral iron as a cause of decompensation of a previously stable disease control [8]. The patient should be informed that the response to iron administration is usually gradual, as opposed to other drugs used in the treatment of the disorder [50]. Even if it would be necessary to measure the brain iron status, in the absence of such a test for routine clinical practice, serum iron status is used to decide if the patient can benefit from iron supplementation. This is done by measuring the serum ferritin concentration and iron binding percentage. If the serum ferritin is ≤75 μg/L or the transferrin saturation is below 20%, iron therapy with oral iron supplements is recommended. It also increases with age: iron supplementation can be considered if the serum ferritin value (in μg/L) is less than the patient’s age [17]. Patients who cannot tolerate oral iron supplements may be considered for iron infusion; high-dose ferric carboxymaltose or low-molecular-weight iron dextran can be used (Table 3) [70,71].

Intravenous iron is also necessary when serum ferritin is not very low, because iron absorption in the intestinal tract is not effective unless there is systemic iron deficiency. Indeed, an endogenous regulatory system exists for oral iron absorption, in physiological conditions, which prevents the patient from excessive serum iron concentration [1,17,70,72].

Daily treatment with drugs for RLS should be started when symptoms are clinically significant, which is when frequency and severity markedly impact on quality of life, such as in the chronic form. Occasional treatment might be considered in intermittent RLS [73].

The most specific therapy for RLS is represented by dopamine agonists: oral drugs, i.e., pramipexole and ropinirole, or skin patches (rotigotine) (Table 3). The main advantage of these drugs is the very fast relief of symptoms, in the short-term period and with low doses, but they are often linked to the development of augmentation, inducing a worsening of disease severity. Low doses, much lower than the ones used for Parkinson disease treatment, are strongly recommended [50,73].

Therefore, in order to prevent augmentation, it has been more recently recommended to start pharmacologic treatment with calcium channel α2δ ligands (pregabalin or gabapentin). Efficacy of 300 mg pregabalin has been demonstrated as compared to pramipexole 0.25 mg, but adverse events are higher with pregabalin [50,73].

For long-term therapy, given the high risk of augmentation with dopaminergic drugs, a calcium channel α2δ ligand is often preferred [50,73].

For a correct therapy, it is essential to respect the correct timing of the medications, according to their pharmacokinetics and the time of occurrence of symptoms. Regarding dopamine agonists and α2δ ligand, the drugs have to be taken generally 1–3 h before symptom onset time (Table 3) [17,50].

For most severe cases, opioids can be used, even if pain is not reported by the patient [50].

Benzodiazepines (clonazepam) are commonly used, although without a specific pharmacodynamic rational, apart from the sedative effect and the associated anxiety relief [50,73].

Especially for the treatment of RLS during pregnancy, nonpharmacologic treatments may play an important role, in order to avoid drug administration. Moderate to intense physical activity should be encouraged, avoiding sports that may expose the pregnant women to the risk of abdominal trauma and recommending not to practice the exercise close to bedtime because this would interfere with sleep and might worsen RLS symptoms [11,74].

If drug therapy is necessary, oral or intravenous iron supplementation should be preferable, if ferritin levels are below 75 µg/L. When dopaminergic drugs are considered the necessary option, the use of carbidopa/levodopa at the daily dose of 20/100 mg up to 50/200 mg is recommended. If the extended-release formulation is used, the risk of augmentation is reduced. Dopamine agonists are contraindicated during pregnancy. Lactation may be inhibited by dopaminergics because of prolactin inhibition, but the effect is rapidly reversible after drug discontinuation [11,74].

Benzodiazepines (clonazepam) can be safely used during the second and third trimesters of pregnancy at low dosages (0.25–1 mg once daily). During the first trimester, a low risk of orofacial malformations has been described [11,74].

Alpha-2-delta ligands and opioids are generally not recommended during pregnancy, but they may be used in most severe and resistant cases. Nonetheless, if necessary, they should be prescribed only during the second and third trimesters, because of teratogenicity (especially congenital heart disease for opioids), although also later in pregnancy, they expose the newborn to the risk of sudden infant death syndrome or neonatal abstinence syndrome [11,74].

## 9. Augmentation

Augmentation, first described by Allen and Earley in 1996 [75], is a phenomenon characterized by: (1) an anticipation of the time of appearance of the RLS symptoms compared with the onset time before starting the medication; (2) a shorter latency of symptoms at rest; (3) a spread of symptoms to other parts of the body; (4) or a greater intensity of the symptoms. Another typical feature of augmentation is the paradoxical effect on RLS symptoms after increasing the dose, with symptom worsening, and improvement after dose reduction (Table 4) [50,76].

Augmentation is typically observed in dopaminergic drugs but is most frequent in levodopa (up to 73%) and less in dopamine agonists. For dopamine agonists, it tends to increase over time, being from less than 10% in the short-term therapy to 42%–68% after 10 years of treatment [50,76]. Short-acting dopamine agonists (ropinirole and pramipexole) are more associated with augmentation compared to longer acting dopamine agonists (long acting oral dopamine agonist or transdermal rotigotine). The latter allows for a lower circadian fluctuation of serum dopamine levels, hence for a less pulsatile stimulation of dopamine receptors, with a reduction in the circadian variations of dopamine receptors’ downregulation [1,17,50,73]. Nonetheless, it cannot be ruled out that the continued exposure to a dopamine agonist over 24 h simply masks instead of treating the augmentation [77].

Differential diagnosis and correct identification of augmentation is important. It evolves slowly over time and may be unrecognized. The main alternative conditions to rule out are: natural waxing and waning course of RLS, other causes of RLS progression such as iron deficiency/low ferritin levels, poor RLS medication compliance, use of RLS exacerbating medications (e.g., antihistamines, selective serotonin reuptake inhibitors), lifestyle changes (e.g., immobilization after bone fracture or surgery), and other physiologic or comorbid conditions (e.g., pregnancy and renal failure) [50].

Moreover, natural progression of RLS, tolerance and end-of-dose exacerbation must be distinguished from augmentation: tolerance is loss of effectiveness of medications over time but does not cause an earlier onset of symptoms; end-of-dose exacerbation (or medication rebound) of symptoms is related to the pharmacokinetic properties of the drug, therefore it manifests as worsening of symptoms at the expected time according to the half-life, commonly in the early morning [17,50].

Management of augmentation and of drug resistant RLS, which are often coexisting conditions, can be done in multiple steps or by an abrupt therapy modification, depending on the severity of symptoms [78]. In mild augmentation, with mild to moderate distress and when the patient is taking dopamine agonists, the clinician can consider advancing the same dose earlier or to split it. A slight dose increase may be considered, but maintaining the minimum dose is recommended.

If not effective, a switch from a short-acting to a long-acting dopamine agonist or to an α2δ ligand should be done. In severe augmentation, a 10-day wash out should be attempted, if possible, and this could significantly relieve symptoms. Alternatively, a switch from a short to a long-acting dopamine agonist or a fast cross titration to an α2δ ligand is necessary. In most severe cases, opioids are recommended or intravenous iron infusion, if ferritin is below 100 µg/L [71,78].

## 10. Conclusions

In the field of chronic diseases and connected risk factors, particular attention has been recently given to the role of sleep health. A clear link between disordered sleep and chronic conditions such as type 2 diabetes, cardiovascular disease, obesity, and depression has been identified [79,80]. The pathophysiological relationship between sleep disorders and chronic diseases has been increasingly explored and the importance of a correct diagnosis and treatment of sleep problems is more and more stressed [6,12,22,25,26,68], in order to prevent premature death and illness. Sleep disorders have direct and expected consequences, regarding both psychic and physical health, as well as indirect consequences, related to healthcare and welfare costs and professional efficiency [68].

Among sleep disorders, RLS represents one of the most common [15] and with a significant impact on many aspects of daily life. It is still underdiagnosed, also in neurological settings, despite the presence of peculiar symptoms reported by the patients [4]. Considering that the diagnosis is based essentially on subjective symptoms, an accurate and detailed history collection is mandatory, in order to avoid misdiagnosis. Instrumental examinations may help for discerning more complex cases or to rule out the disease [18]. RLS mimics may indeed be challenging and, in order to achieve an effective management, the first step is proper diagnostic work-up [23,24].

RLS has typically a chronic evolution, thus affecting a broad span of a patient’s lifetime and, on the other hand, altering the health status of patients from different age ranges and of both sexes [15,17].

The diagnosis of RLS can be challenging in some patients, especially children and elderly people [16,18,60], and the effective treatment might be difficult to obtain, because of problems inherent to the disease itself and to its specific features as well as of certain conditions characterizing different patient groups [69,73,74,75,76,77,78]. Comorbidities can appear overtime and complicate the disease manifestations or can represent the main cause of the symptoms [1,6,17]. On the other hand, some special life conditions, such as pregnancy, can be distinctly associated with the disease, contributing to a better knowledge of the disease mechanisms and posing the need for special attention in terms of therapeutic management [11,58,59,74].

Disease natural progression, drug tolerance and augmentation represent the main problems related to the disease management, developing overtime and asking for careful evaluation by the clinician. Drug therapy of RLS may necessarily change across the lifespan of a patient, requiring specific adjustments in specific conditions [1,17,73,78].

The early stages of RLS are commonly characterized by the occurrence of symptoms only at night and/or in the evening and by a good response to first-line treatments. When symptoms occur both in the morning and at night, more complex or multifactorial pathophysiology might be suspected, and less effectiveness of drug treatment can be observed. Therefore, although it is not univocal to define these two conditions as two different diseases, the possibility that they represent two extremes of a severity range or two distinct phenotypes of the same disease might be taken into account. This can be related to disease progression if it occurs overtime, or to more severe pathophysiological alterations if it represents the initial pattern of the disease presentation.

The most recent insights on RLS pathophysiology give a remarkable contribution to the treatment of the disease and shed light on the complex environmental and genetic interactions at the base of this disease, which frequently underlies a familial predisposition [1,8,29,30,31,32].

Finally, the correct diagnosis and management of RLS, as well as of other sleep disorders, have notable and favorable effects in terms of the quality of life of the patients and of their professional and social functioning [68].

## Figures and Tables

**Table 1:** International Restless Legs Syndrome Study Group (IRLSSG) consensus diagnostic criteria [18].

**Essential diagnostic criteria (all must be met):**
An urge to move the legs usually but not always accompanied by, or felt to be caused by, uncomfortable and unpleasant sensations in the legs. ^a,b^ The urge to move the legs and unpleasant sensations begin or worsen during periods of rest or inactivity such us lying down or sitting. The urge to move the legs and unpleasant sensations are partially or totally relieved by movement, such as walking or stretching, at least as long as the activity continues. ^c^ The urge to move the legs and any accompanying unpleasant sensations during rest or inactivity only occur or are worse in the evening or night than during the day. ^d^ The occurrence of the above features is not solely accounted for as symptoms primary to another medical or a behavioral condition (e.g., myalgia, venous stasis, leg edema, arthritis, leg cramps, positional discomfort, habitual foot tapping).
**Supportive criteria:**
Family history of RLS/WED among first-degree relatives. Dopaminergic treatment response. Periodic leg movements during wakefulness or sleep. Lack of profound daytime sleepiness.

^a^ the urge to move is essential and it is sufficient for the diagnosis, unlike the unpleasant sensations in the legs, which are neither sufficient nor necessary for RLS/WED diagnosis. RLS/WED may involve other body parts. ^b^ for children, the description of these symptoms should be in the child’s own words. ^c^ in severe RLS/WED relief by activity may not be noticeable but must have been previously present. ^d^ in severe RLS/WED, the worsening in the evening or night may not be noticeable but must been previously present.

**Table 2:** Differential diagnosis of RLS (RLS mimics) [18,23,24].

Disorder	Clinical Features
Common mimics	
Neuropathic pain syndrome	Not relief with movement
Peripheral neuropathy	Gloves and stocks distribution
Radiculopathy	Radicular pain distribution Weakness Antalgic position
Sleep related leg cramps	Palpable tightening of the muscle Alleviated by stretching and not by movement
Positional discomfort	Relief with postural shift No circadian pattern
Venous stasis	Leg edema Skin alterations
Arthritis	Symptoms confined to a joint, joint erythema Delayed relief with movement
Anxiety	Mainly psychic symptoms Volitional movements of the legs, without sensory discomfort
Habitual foot tapping	No urge to move, reduced awareness of the movement
Drug-induced akathisia	Neuroleptic assumption Body rocking movements Whole body involvement
Less common mimics	
Myelopathy	Hypoesthesia Muscle atrophy, weakness
Myopathy	No circadian pattern Proximal distribution, cramps Muscle atrophy, weakness, skin alterations
Vascular or neurogenic claudication	Relief at rest Increase during legs movements No circadian pattern Skin alterations
Hypotensive akathisia	No circadian pattern Increase during sitting position Relief in lying position
Painful legs and moving toes	No circadian pattern No sensory discomfort
Vesper’s curse	Lumbosacral pain Cardiopulmonary alterations
Pediatric mimics	
Growing Pain	Relief with local massage and stretching
Attention-deficit hyperactivity disorder	Inattention, hyperactivity, disruptive behavior, and impulsivity

**Table 3:** Treatment of restless legs syndrome in adult patients [1,17,71,73].

Medication	Daily Starting Dose	Effective Daily Dose (Maximum Recommended Daily Dose—MDD)	Suggested Timing of Drug Intake	FDA/EMA Approved for RLS	Notes
***α2δ ligands***					
Gabapentin	300 mg (<65 y) 100 mg (>65 y)	300–2400 mg (MDD not defined)	2–3 h before symptom onset	No	-pharmacokinetic limitations -may need multiple doses
Gabapentin enacarbil	600 mg (<65 y) 300 mg (>65 y)	300–1200 mg (MDD 600 mg)	2–3 h before symptom onset	FDA	-taken with food
Pregabalin	75 mg (<65 y) 50 mg (>65 y)	75–450 mg (MDD not defined)	-	No	-no augmentation
***Dopamine agonist/Levodopa***					
Pramipexole	0.125 mg (salt) 0.088 mg (base)	0.125–0.75 mg (salt) (MDD 0.75 mg) 0.088–0.54 mg (base) (MDD 0.54 mg)	1–3 h before symptom onset	Yes	-augmentation -better PLMS reduction
Ropinirole	0.25 mg	0.25–4 mg (MDD 4 mg)	1–3 h before symptom onset	Yes	-augmentation -better PLMS reduction
Rotigotine patch	1 mg	1–3 mg (MDD 3 mg)	1–3 h before symptom onset	Yes	-augmentation
Levodopa/carbidopa Levodopa/benserazide	100/25 mg: ½ or 1 tab	100/25 mg: 1–3 tab (MDD not defined)	-	No	-intermittent RLS -high risk augmentation
***Opioids***					
Oxycodone/naloxone prolonged release	5 mg oxycodone/2.5 mg naloxone: twice daily	10–20 mg oxycodone/ 5–10 mg naloxone: twice daily (MDD 40 mg oxycodone/20 mg naloxone: twice daily)	-	EMA for second line therapy	-addiction and dependency uncommon
Codeine	15–30 mg	15–120 mg (MDD not defined)	-	No	-addiction and dependency uncommon
Methadone	2.5 mg	5–40 mg (MDD not defined)	-	No	-addiction and dependency uncommon
***Iron supplementation***					
Intravenous Ferric carboxymaltose	1000 mg	1000 mg in one infusion (MDD not defined)	-	No	-ferritin <100 µg/L and transferrin saturation <45% -allergic reactions
Intravenous Iron sucrose	200 mg	200 mg in five infusions (MDD not defined)	-	No	-ferritin <100 µg/L and transferrin saturation <45% -allergic reactions
Ferrous sulfate and vitamin C	325 mg ferrous sulfate and 200 mg vitamin C	325 mg ferrous sulfate: twice daily and 200 mg vitamin C (MDD not defined)	-	No	-ferritin <75 µg/L and transferrin saturation <20% -empty stomach
***Benzodiazepine***					
Clonazepam	0.25 mg	0.25–2 mg (MDD not defined)	-	No	

For treatment during pregnancy and lactation, referring to specific guidelines [74].

**Table 4:** Max Planck Institute diagnostic criteria for augmentation [76].

***Basic features augmentation***: increase in symptom severity after initial therapeutic benefit, which is not accounted for by other factors. Increase in symptom severity was experienced on five out of seven days during the previous week.
In addition, either A or B or both have to be met:
paradoxical response to treatment (increase severity of symptoms increasing medication and an improvement following decrease in medication). Earlier onset of symptoms by at least 4 h
or
Earlier onset of symptoms between 2 h and 4 h with one of the following compared to symptom status before treatment:
◦ Shorter latency at rest ◦ Extension of symptoms to other body parts ◦ Greater intensity of symptoms * ◦ Shorter duration of relief from treatment

* or increase in periodic limb movements if measured by polysomnography or the suggested immobilization test.
